# Chronic Analgesic Nephropathy with Atypical Squamous Metaplasia

**DOI:** 10.7759/cureus.83400

**Published:** 2025-05-03

**Authors:** Lee P Mannion, Kyle A Davis, Devan Makati, Bronson Herr, Shahrier Amin

**Affiliations:** 1 Department of Business Administration, Knauss School of Business, University of San Diego, San Diego, USA; 2 Department of Nephrology, West Virginia University School of Medicine, Morgantown, USA; 3 Department of Nephrology, West Virginia University Hospital, Morgantown, USA; 4 Department of Surgery, West Virginia University School of Medicine, Morgantown, USA; 5 Department of Pathology, West Virginia University School of Medicine, Morgantown, USA

**Keywords:** chronic analgesic nephropathy, kidney injury, nonsteroidal anti-inflammatory drugs, oncology, squamous metaplasia

## Abstract

Nonsteroidal anti-inflammatory drugs (NSAIDs) are a mainstay of pain management for various conditions. Their chronic use is associated with a well-documented risk of chronic analgesic nephropathy. Squamous metaplasia, a rare sequela of chronic analgesic nephropathy, involves the transformation of the normal epithelial lining of renal tubules into squamous epithelium. This metaplasia poses a diagnostic challenge, as it can mimic malignancy due to its atypical features. We present a case of a 64-year-old male patient with fibromyalgia and chronic NSAID use who developed chronic analgesic nephropathy concerning for squamous metaplasia, highlighting the potential for severe renal complications associated with chronic NSAID use.

A kidney biopsy was evaluated using light microscopy, immunofluorescence microscopy, and electron microscopy. The kidney biopsy revealed histological features characteristic of chronic analgesic nephropathy, including severe interstitial fibrosis and tubular atrophy. Additionally, extensive squamous metaplasia involving the renal tubules was identified at the glomerular level in the absence of a primary mass. This metaplasia exhibited worrisome features such as atypia and increased mitotic activity, signifying a heightened rate of cell division. This case report underscores the potential for chronic NSAID use to induce significant kidney damage, culminating in chronic analgesic nephropathy and squamous metaplasia with atypical characteristics. Integrating various microscopy techniques supports a definitive diagnosis and guides patient management.

## Introduction

The alarming increase in chronic kidney disease (CKD) demands a closer look at all potential causes, especially those that can be prevented. Analgesic nephropathy, a type of CKD caused by long-term use of pain relievers like ibuprofen or naproxen (nonsteroidal anti-inflammatory drugs (NSAIDs)), is a significant contributor to this growing health problem. While doctors are familiar with the typical signs of analgesic nephropathy, a rare complication like squamous metaplasia makes this diagnosis difficult.

This case study examines a 64-year-old man with fibromyalgia who developed analgesic nephropathy along with this unusual complication. By carefully examining the detailed tissue samples from his kidney biopsy, we aim to understand how squamous metaplasia might develop in analgesic nephropathy and improve the way doctors diagnose this challenging condition. The significance of this case extends beyond a single patient and has the potential to inform future medical practices and potentially lead to better diagnostic tools and treatments for analgesic nephropathy with squamous metaplasia.

## Case presentation

A 64-year-old male patient presented to our facility for a kidney biopsy due to significantly elevated creatinine levels of 7.6 milligrams per deciliter (mg/dL). Past medical history was significant for hyperlipidemia, hypertension, pre-diabetes, chronic obstructive pulmonary disease (COPD), and fibromyalgia. The patient was a chronic smoker (two packs per day for 50 years) and was a long-term user of NSAIDs for his fibromyalgia.

Laboratory findings showed elevated serum creatinine (1.2 mg/dL in September 2023 to 9 mg/dL in March 2024), which confirmed a rapid decline in kidney function. Urinalysis showed proteinuria of 30 mg/dL. Normal complement levels were C3 129 mg/dL and C4 47 mg/dL, as well as a free light chain ratio of 1.53 mg/dL, which argued against immune complex or paraprotein-related kidney diseases, respectively.

Based on the above findings, a renal biopsy was performed. Light microscopy assessed different aspects of the kidney tissue. Sections submitted for light microscopic examination contained 70% renal cortex and 30% renal medulla. Hematoxylin and eosin (H&E) staining likely revealed the overall architecture of the kidney, including the size and cellularity of glomeruli, the presence of scarring (fibrosis), and tubular atrophy. Up to six glomeruli were present, none of which were globally sclerotic. The glomeruli were of normal size and cellularity. Most glomeruli showed wrinkling and ischemic changes.

**Figure 1:**
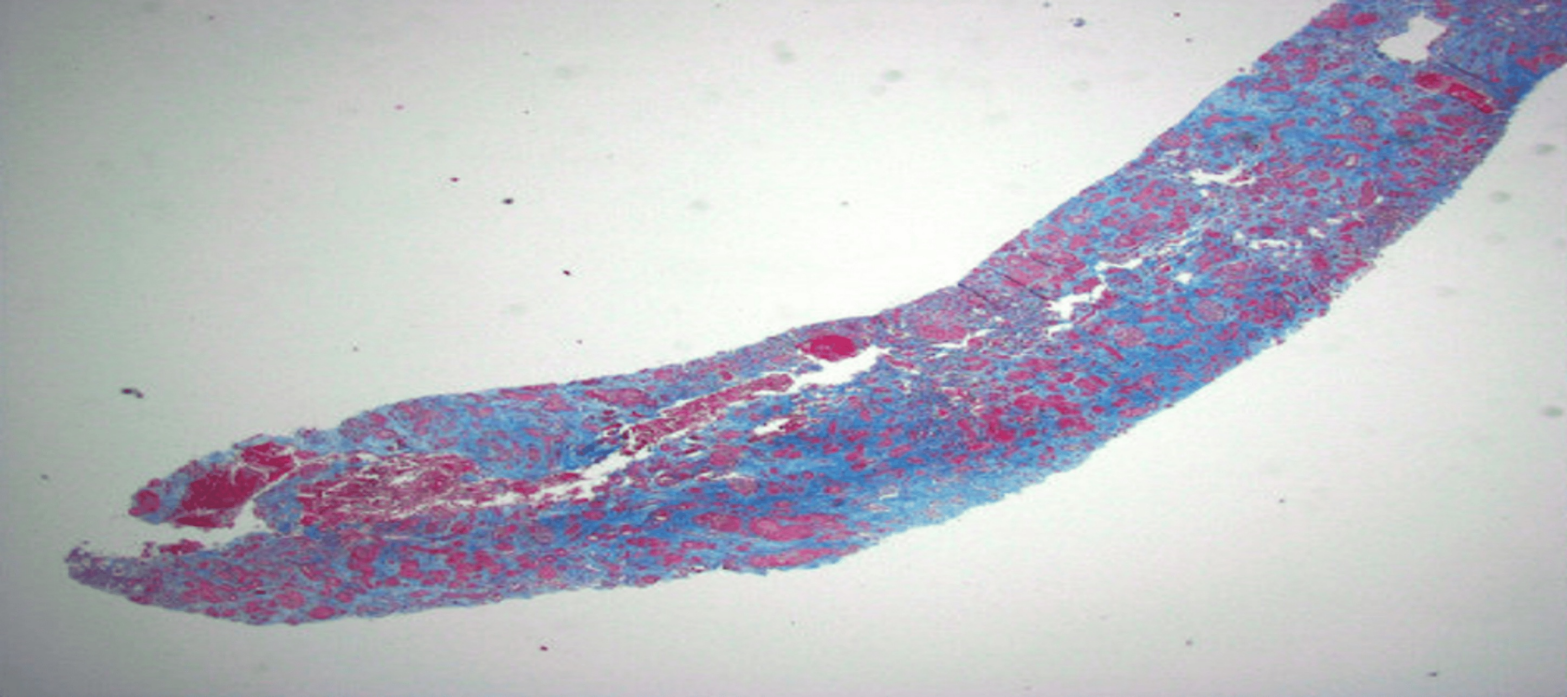
Low-power light microscopy (trichrome stain, 20x) showing severe fibrosis and atrophy of the renal cortex

The most striking finding was moderate-to-severe interstitial fibrosis and tubular atrophic changes in 40% to 60% of the cortex observed in both H&E stains and trichome stains, indicating significant damage to the nephrons, the functional units of the kidney responsible for filtration (Figure 1).

**Figure 2:**
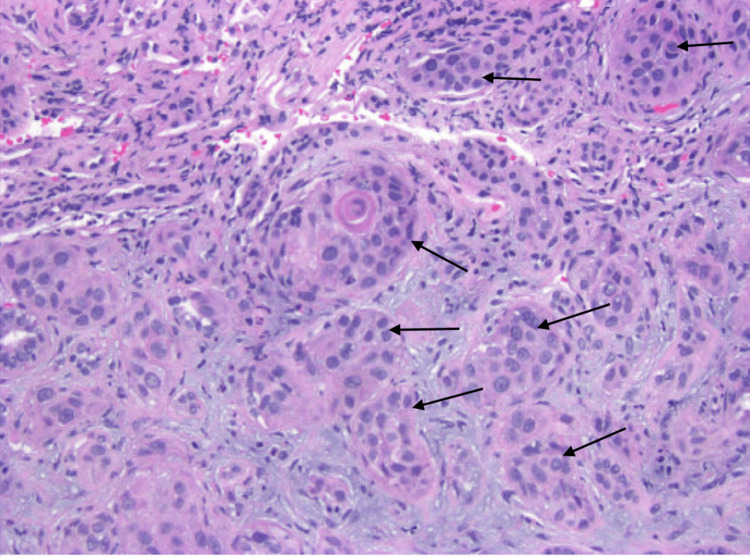
High-power light microscopy (hematoxylin and eosin stain, 400x) showing squamous metaplasia with atypia (arrows)

The renal medulla showed coagulative necrosis. Extensive metaplastic changes were noted, with predominantly squamous features and focal areas of urethral-type transformation. Keratohyalin granules and keratinization were also observed, though focal, once again, indicating the replacement of normal urothelium. Some of the metaplastic areas showed atypia with pleomorphism and increased mitotic activity (Figure 2). Periodic acid-Schiff (PAS) and silver stains showed thickened basement membranes, which can also impair the filtration process and indicate kidney damage.

Immunoperoxidase stains were used to exclude infectious causes. Cytomegalovirus (CMV), BK virus, and herpes simplex virus (HSV) were negative, ruling out these as potential causes of kidney damage. However, we confirmed metaplastic changes with positive staining for p40, CK7, and GATA3 in the atypical areas.

**Figure 3:**
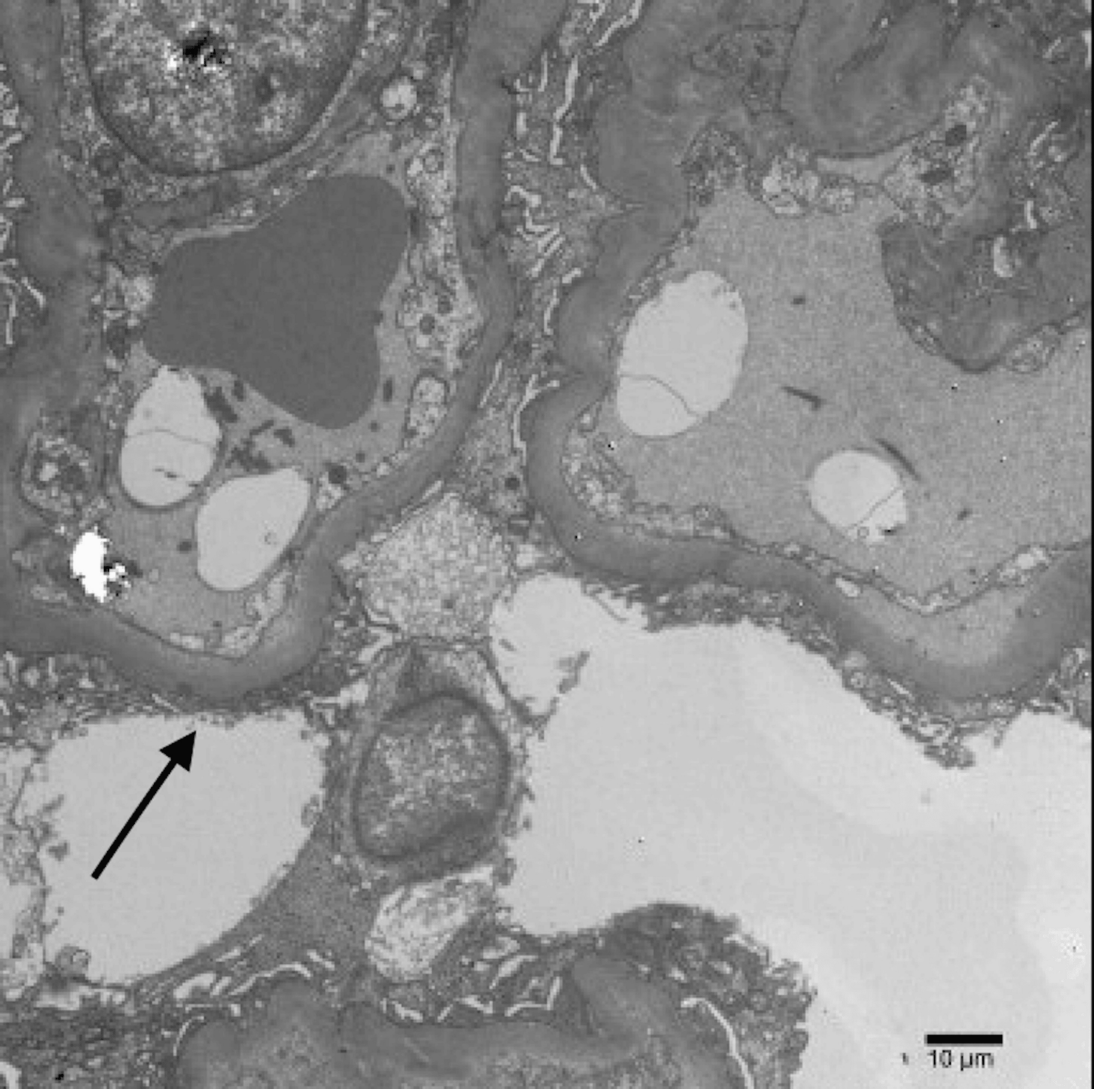
Transmission electron micrograph showing thickened glomerular basement membranes and severe foot process effacement

Electron microscopy revealed patent capillary loops. The glomerular capillary basement membranes were thickened to two to three times normal. There was diffuse and severe foot process effacement of the podocytes with microvillous transformation (Figure 3). No immune-type electron-dense deposits were seen along the glomerular capillary loops or in the mesangial areas. Immune-mediated injury is more characteristic of membranous nephropathy and membranoproliferative glomerulonephritis; however, such a mechanism is not supported by the findings in this case. The tubules show marked attenuation of the brush border, and the tubular basement membranes were without deposits.

Sections submitted for immunofluorescence microscopy contained the renal cortex. Up to 12 glomeruli were present, one of which was sclerotic. The sections were stained with IgA, IgG, IgM, C3, C1q, albumin, fibrinogen, kappa light chain, and lambda light chain. All stains were negative in the glomeruli and tubulointerstitium.

The patient's history of chronic use of anti-inflammatory drugs, including NSAIDs, for fibromyalgia is a known cause of tubulointerstitial nephritis and likely contributed to the observed tubular atrophy, interstitial fibrosis, and basement membrane thickening. NSAIDs are also known to cause renal papillary necrosis and changes reminiscent of minimal change disease with severe foot process effacement. Altogether, the changes seen in this biopsy are favored to be due to chronic analgesic nephropathy as the primary cause of the patient’s declining kidney function. There was no evidence of a glomerular disease of the immune complex type or a paraprotein-related disease. Importantly, the squamous metaplastic areas in the biopsy showed increased mitotic activity and atypia. Computed tomography (CT) of the abdomen and pelvis without intravenous (IV) contrast revealed no findings of a primary renal mass.

The patient's smoking history was another risk factor for kidney disease, and its potential contribution cannot be entirely ruled out. Stopping NSAID use is crucial to prevent further kidney damage. Alternative pain management options for fibromyalgia should be explored with the doctor to include tricyclics and selective serotonin-norepinephrine reuptake inhibitors. Close follow-up with regular blood tests, urinalysis, and potentially repeat biopsies in the future will be necessary to monitor kidney function and the atypical squamous metaplasia. Management of underlying conditions such as hypertension and pre-diabetes can mitigate kidney disease progression. Optimizing their management through medication or lifestyle changes can help protect remaining kidney function.

The severity of chronic analgesic nephropathy determines the long-term outlook. The extent of kidney damage observed in the biopsy suggested a significant decline in function. Early detection and stopping NSAIDs can help prevent further damage and potentially stabilize or even improve kidney function to some extent. The presence of atypical squamous metaplasia introduced uncertainty. Close monitoring is essential to assess if the atypical cells progress towards malignancy. Early detection and treatment of potential cancer can significantly improve the prognosis.

The patient’s care in this case was further managed by oncology for metastatic squamous cell carcinoma of unknown primary. Single-agent immunotherapy with pembrolizumab was recommended. Side effects were discussed with the patient. Of note, he had multiple emergency room visits over his treatment course for incidental hypercalcemia found on lab work. The patient suffered a pathological pelvic fracture during the duration of his treatment. The patient ultimately passed away three months post-nephrology findings from metastatic disease.

## Discussion

The kidney biopsy findings present a diagnostic challenge. While the clinical presentation and histological features strongly suggest chronic analgesic nephropathy, the atypical squamous metaplasia necessitates further exploration. Squamous metaplasia within the kidney is an uncommon incidental finding during biopsy procedures. This is because it often presents without the classic clinical signs or laboratory abnormalities that would typically warrant a biopsy in the first place.

Our case exemplifies this. The initial biopsy was prompted by a clinical picture suggestive of tubulointerstitial nephritis, likely secondary to chronic NSAID use. The incidental finding of atypical squamous metaplasia raises the concern for a potential malignancy deviating from the usual histopathological presentation of primary renal neoplasms. While renal cell carcinoma is the most common malignant kidney tumor [1], squamous cell carcinoma arising from metaplasia represents a rare fraction of such incidental findings [2].

Traditionally, predisposing factors for squamous metaplasia include chronic infections and renal stones [2]. Over time, these factors can compromise the integrity of the underlying renal epithelium, promoting squamous transformation. Reported cases of renal squamous metaplasia often correlate with renal calculi, leading to hydronephrosis, calcification, and regional lymph node enlargement [3]. Our patient, however, lacked these characteristic presentations.

In contrast, their chronic NSAID use aligns with the typical renal complications observed in the biopsy, including inflammatory infiltrates. This presentation aligns with a traditional biopsy performed to confirm tubulointerstitial nephritis secondary to chronic NSAID use.

Other chronic inflammatory conditions, like xanthogranulomatous pyelonephritis or tuberculosis, can occasionally mimic squamous cell carcinoma of the kidney, but their clinical presentations differ from our case [4]. Xanthogranulomatous pyelonephritis, a rare chronic bacterial infection caused by organisms like *Proteus mirabilis* and *Escherichia coli*, often presents with large, irregular masses visible by radiology on the kidney, which were absent in our patient [4-6].

Therefore, primary or urothelial-derived renal squamous cell carcinoma is a rare entity. It is classically associated with renal stones, making our patient's presentation without these typical predisposing factors rather unique [7,8]. This ambiguity raises intriguing questions on whether the squamous metaplasia might have originated from a distant, unidentified source or if it might be a rare presentation of a primary renal neoplasm incidentally discovered during a biopsy for an unrelated diagnosis in the absence of a primary renal mass [9-13].

## Conclusions

This case report highlights the potential for chronic NSAID use to culminate in severe kidney injury, including chronic analgesic nephropathy, concerning for atypical squamous metaplasia. The atypical features of the squamous metaplasia necessitate close monitoring for potential malignant progression. This case also underscores the importance of integrating various microscopy techniques for a comprehensive diagnosis, especially when atypical presentations arise.

The rarity of squamous metaplasia in the absence of predisposing factors like renal calculi warrants further investigation into the potential mechanisms by which chronic NSAID use might contribute to this complication. Additionally, future research could explore the utility of specific biomarkers to differentiate between benign and malignant squamous metaplasia in such cases. From a clinical standpoint, this case emphasizes the importance of considering atypical squamous metaplasia as a differential diagnosis in patients with chronic NSAID use who present with declining kidney function, even in the absence of typical risk factors.
